# Efficacy and Safety of N-Butyl 2 Cyanoacrylate Injection for Treatment of Gastric Varices: A five year experience from a tertiary care hospital in Karachi, Pakistan

**DOI:** 10.12669/pjms.38.5.4999

**Published:** 2022

**Authors:** Nazish Butt, Farhan Haleem, Muhammad Ali Khan, Amanullah Abbasi

**Affiliations:** 1 Dr. Nazish Butt, MBBS, FCPS, MACG. Assistant Professor, Department of Gastroenterology, Jinnah Postgraduate Medical Centre, Karachi, Pakistan, Agha Khan University Hospital, Karachi, Pakistan; 2 Dr. Farhan Haleem, MBBS, FCPS. Jinnah Postgraduate Medical Centre, Karachi, Pakistan; 3 Dr. Muhammad Ali Khan, MBBS. Jinnah Postgraduate Medical Centre, Karachi, Pakistan; 4 Prof. Amanullah Abbasi, MBBS, FCPS, MRCP. Dow University of Health Sciences, Karachi, Pakistan

**Keywords:** Efficacy, Safety, Gastric varices, N-Butyl 2 Cyanoacrylate

## Abstract

**Objectives:**

To evaluate the efficacy and safety of N-Butyl 2 Cyanoacrylate injection for treatment of gastric varices.

**Methods:**

This was a retrospective observational cohort conducted at Medical Unit IV, Department of Gastroenterology, JPMC, Karachi from January 2014 to December 2018 (five years). All patients irrespective of age and gender that presented to the emergency department with complain of hematemesis or stigmata of UGIB were potential candidates. Once they were resuscitated and endoscopic evidence of gastric varices requiring intervention was found, the patients were inducted into the study. N-Butyl 2 Cyanoacrylate injection was performed until hemostasis and obliteration of gastric varices was achieved. Response to therapy was analyzed at three weeks with repeat endoscopy. Mortality was analyzed at six weeks. Major and minor complications recorded as well.

**Results:**

A total of 159 patients were inducted into the study. Isolated gastric varices Type-I was mostly encountered. A singular session was sufficient to achieve obliteration and hemostasis in over three quarter of the patients. Complications affected 11.30% of the patients. Six weeks mortality was exceptionally low at 1.20%.

**Conclusions:**

N-Butyl 2 Cyanoacrylate is safe and effective for treatment of gastric varices. There were few complications seen with this procedure in this study. It significantly reduces mortality at six weeks.

## INTRODUCTION

Upper gastrointestinal bleeding (UGIB) is encountered at the emergency rooms consistently throughout the world. Mortality rates of up to 10% have been seen, these rates are higher with variceal bleeding.^1^ Higher rates of variceal bleeding are seen in regions where chronic viral hepatitis is endemic with corresponding rates of cirrhosis^2^ such as Pakistan.

Variceal bleeding requires urgent endoscopic intervention despite the fact that a quarter of bleeding will subside spontaneously and a higher percentage will respond to pharmacotherapy alone. Endoscopic variceal band ligation (EVBL) is the treatment of choice. However, not all bleeds in cirrhosis are due to esophageal variceal bleeding. Other sources include portal hypertensive gastropathy, stress ulcers and gastric (fundal) varices.

Of these, gastric varices contribute to the highest rates of morbidity and mortality. The prevalence of gastric varices is 17% to 70% in portal hypertension.^3^ Gastric varices are associated with increased rebleeding rates and mortality.^4^ The gold standard of treatment is N-Butyl 2 Cyanoacrylate injection into varices leading to obliteration of the vessel(s).

Classification of gastric varices was first proposed by Sarin et al.^3^, the varices are classified according to their anatomical location and their communication or lack thereof with the esophageal varices. Gastro-esophageal varices 1 (GOV1) are esophageal varices extending down to the cardia or lesser curvature of the stomach. Gastro-esophageal varices 2 (GOV2) are esophageal varices extending down to the greater curvature or fundus of the stomach. Isolated gastric varices 1 (IGV1) are located in the fundus without any communication to the esophageal varices if they are present. Isolated gastric varices 2 (IGV2) are located elsewhere in the stomach, without any communication to the esophageal varices if they are present. It is not uncommon to find a combination of any of the four variants described above.

N-Butyl 2 Cyanoacrylate injection is a relatively inexpensive and easy to perform procedure with good outcomes.^5^ The procedure is associated with complications such as rebleeding, infection, sepsis, thromboembolic events, perforation and recurrent bleeding. Factors contributing to worse outcomes and complications are increasing or large size of varices, advanced liver disease (high Child Pugh score), co morbids, organ failure and malignancy.

N-Butyl 2 Cyanoacrylate is an adhesive/glue, it polymerizes (solidifies from a liquid state) very quickly^6^. It is used for sclerotherapy of large esophageal or gastric varices, closure of skin wounds and in fixation of mesh for inguinal hernia surgery. It comes in two colors, blue and transparent. It is easy to use and has few adverse effects.

The principle of this technique is to inject the glue into the varix, which then solidifies causing a tamponade effect. This in turns blocks the bleeding and leads to obliteration of the vessel over time. At times more than one procedure is needed for complete vessel obliteration. This procedure has the following steps.

N-Butyl 2 Cyanoacrylate injection was performed using a GIF-HQ 190 or a GIFQ 180 endoscope (Olympus C, Tokyo, Japan) and 23-gauge disposable injection needle catheter (1650 mm in length). Each shot contained 1.0 cc N-Butyl 2 Cyanoacrylate and 1-1.5cc of Lipiodol^6^ (Guerbet, Aulnay-sous-Bois, France). The endoscopic suction channel was flushed with lubricant before insertion of the catheter.

The preload sclerotherapy catheter was advanced through the endoscope into the stomach, and the needle was inserted directly in the gastric varix. Lipoidal was given through the catheter to deliver the glue mixture into the varix, and the needle was then withdrawn while glue was still flowing to decrease the risk of needle embedment. A good number of the patients also have esophageal varices that bleed concomitantly or require intervention. In such cases band ligation was performed as well.

In this first study of its nature conducted at Jinnah Postgraduate Medical Centre. We have analyzed the safety and efficacy of N-Butyl 2 Cyanoacrylate injection for treatment of gastric varices. The prevalence of gastric varices, various etiologies, co morbids, types of gastric varices and their effect on outcomes was also assessed.

## METHODS

**Fig.1:**
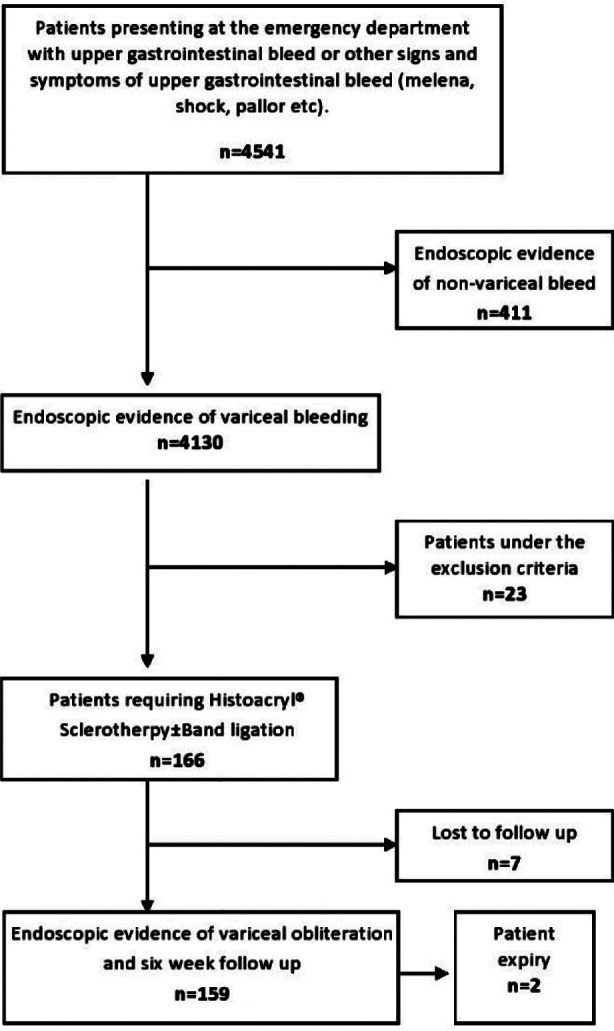
Flow chart of patient included in the study.

This was a retrospective observational cohort conducted from January of 2014 to December of 2018 (five years). It was held at the Medical Unit IV, department of Gastroenterology. The study was approved by the institutional review board committee of JPMC, NO.F.2-81-IRB/2018 GENL/9386/JPMC. Patients presenting with hematemesis and/or melena were first resuscitated at the emergency room. Once the patients were hemodynamically stable, they were admitted to the intensive care unit (ICU). Endoscopy was carried out as soon as possible. Patients in whom gastric varices were identified and required N-Butyl 2 Cyanoacrylate injection sclerotherapy to achieve hemostasis were inducted into the study (Fig.1). Relevant work up was carried out before and after the procedure to ensure patient optimization.

Patients were excluded from the study if other additional identifiable sources of bleeding other than gastric varices were found on endoscopy, gastric varices that did not warrant intervention and cases complicated severe debilitating diseases even if Histoacryl injection was required.

### Primary Outcome:

First end point was the obliteration of the fundal varices; whether obliteration required a single session or multiple sessions was also analyzed. Of course, a successful procedure was associated with control of bleeding initially. Obliteration was confirmed via repeat endoscopic procedures, defined as absence of any identifiable vessels (veins) that may cause future bleeding or having stigmata of recent bleed after intervention. In case of continued bleeding, repeat sclerotherapy was performed on emergency basis.

Repeat endoscopy was performed at three weeks after initial hemostasis achievement. If there were no gastric varices seen, the patient was followed up as per guidelines. If repeat sclerotherapy was required then it was done then and there; yet another endoscopy was performed again at three weeks to seek obliteration. This process was carried out as many times as required.

### Secondary Outcomes:

There were two secondary end points. One outcome was the six weeks mortality after complete obliteration of the varices. Complications during the procedure and after it were also analyzed. Two most frequent complications were “Re-bleed”, defined as bleeding within 24 hours of the procedure and “Recurrent bleeding” defined as bleeding after 24 hours but within seven days of the procedure. Any episode of bleeding that occurred after seven days was considered a new event and not a complication of the procedure. Thirdly non bleeding complications such as fever, abdominal pain, pulmonary embolism, abscess formation, bacteremia and perforation were recorded as well.

### Statistical analysis:

Statistical Package for the Social Sciences (SPSS) version 21.0 software (SPSS Inc., Chicago, IL, USA) was used to analyze data. The descriptive statistics of age, and baselines labs were presented as standard deviation and mean. The demographic variables such as gender, Child Pugh class, etiologies of portal hypertension, different type of gastric varices, number of sessions of sclerotherapy, complications and mortality were presented as frequency and percentage.

## RESULTS

One hundred and fifty nine patients were inducted into the study. The prevalence of gastric varices in bleeders due to cirrhosis (portal hypertension) was 4.01% and in overall cases of upper gastrointestinal bleed it was 3.65% (Fig.1). All lab values are consistently representative of decompensated liver disease. The baseline characteristics of the patients included into the study are shown in Table-I.

**Table I:** Baseline characteristics of patients included into the study.

	N=159
**Gender**	
Male	74 (46.5%)
Female	85 (53.5%)
	Mean ± Standard deviation
Age	52.18±11.58 years
Hemoglobin	8.34±2.15
White blood cell	6.97±4.22
Platelets	103.47±58.92
International normalized ratio (INR)	1.25±0.39
Total Bilirubin	1.73±5.14
Alanine aminotransferase (ALT)	46.55±75.72
Aspartate aminotransferase (AST)	15.42±31.93
Gamma-glutamyl transferase (GGT)	24.52±21.74
Serum Albumin	2.66±0.71
Creatinine	1.02±0.36
Serum Sodium	132.86±19.22
Serum Potassium	3.68±0.84

The majority of cases of portal hypertension were due to chronic viral hepatitis. Most common Child Pugh class was B. Nearly; equal number of male and female patients were included in the study. IGV1 variant of gastric varices was mostly encountered; not all variants were seen. Large esophageal varices were present in 61(38.3%) of the cases mostly with IGV1 type gastric varices. Etiologies of cirrhosis/portal hypertension, severity of liver disease, types of gastric varices and presence of esophageal varices with gastric varices are summarized in Table-II.

**Table II:** Etiologies of cirrhosis, liver disease severity scores, types of gastric varices and relative percentage of esophageal varices.

Etiologies of cirrhosis/portal hypertension	N(%)
Hepatitis C Virus (HCV)	117 (73.1%)
Hepatitis B Virus (HBV)	13 (8.1%)
HBV+HCV	2 (1.3%)
HBV+ Hepatitis Delta Virus (HDV)	2 (1.3%)
Non B, Non C cirrhosis	18 (11.3%)
Alcoholism	3 (1.9%)
Hepatocellular carcinoma	3 (1.9%)
Non cirrhotic portal hypertension	1 (0.6%)
**Child Pugh classification**	
Child Pugh Class A	42 (26.41%)
Child Pugh Class B	104 (65.40%)
Child Pugh Class C	13 (8.17%)
MELD score	11 (median)
**Types of gastric varices**	
Isolated gastric varices 1 (IGV 1)	139 (87.4%)
Isolated gastric varices 2 (IGV 2)	Nil
Gastro-esophageal varices (GOV 1)	Nil
Gastro-esophageal varices (GOV 2)	18 (11.3%)
IGV 1 + GOV 2	2 (1.3%)

Most often a singular session was required for variceal obliteration and hemostasis was achieved in 88.67% of the patients after one session. There were very few complications related to the procedure. The six weeks mortality was exceptionally low as well. The most common bleeding complication encountered was Recurrent bleeding, 18 (11.30%) of the patients developed this. Abdominal pain developed in 14 (8.80%) of the patients, it was mostly mild and resolved spontaneously. Sessions required for variceal obliteration, achievement of hemostasis, complications of the procedure and mortality rates are demonstrated in Table-III.

**Table III:** Number of sessions needed for variceal obliteration, achievement of hemostasis, complications of the procedure and six weeks mortality rates.

	N (%)
**Number of sessions needed for variceal obliteration**	
One session	117 (73.10%)
Two sessions	30 (18.86%)
Three sessions	7 (4.4%)
Four sessions	3 (1.9%)
Overall mean	1.35±0.65
**Sessions needed to achieve hemostasis**	
One session	141 (88.67%)
Two sessions	17 (10.69%)
Hemostasis not achieved (mortality)	2 (1.25%)
**Complications of the procedure**	
Recurrent bleed	18 (11.30%)
Abdominal Pain	14 (8.80%)
Fever	7 (4.40%)
Rebleed	1 (0.6%)
Pulmonary Embolism	Nil
Bacteremia	Nil
Abscess formation	Nil
Perforation	Nil
**6 weeks mortality rate**	
6 weeks survival	157 (98.80%)
Death within 6 weeks	2 (1.20%)

## DISCUSSION

Hematemesis or upper gastrointestinal bleed (UGIB) is a common complain encountered at emergency departments. International data suggests that acid peptic disease or gastro-duodenal ulcers are the most common cause of UGIB.^7^ However, for the Indo-Pak region where chronic viral hepatitis is endemic, esophageal and gastric variceal bleeding have disproportionately high prevalence even in children.^8^ The incidence of the gastric varices in our cohort was however, significantly lower as compared to previous studies; evaluation of the multifactorial reasons impacting this incidence is beyond the scope of our cohort.

Previous studies have demonstrated consistent results with respect to efficacy, safety and number of sessions required for variceal obliteration using N-Butyl 2 Cyanoacrylate intervention in gastric varices.^9^,^10^ Locally, Khawaja A et al. showed that a vast majority of patients presenting with gastric variceal bleeding (over a period of more than a decade) required only a singular session for achievement of hemostasis and variceal obliteration; rebleeding was observed in a quarter of patients in his cohort.^10^ These results are quite comparable to what we have recorded. Kamani L et al. and Mumtaz K et al. have also reported similar outcomes locally.^11^,^12^

Internationally, even higher rates of successful control of bleeding, variceal obliteration with lower rates of rebleeding using N-Butyl 2 Cyanoacrylate sclerotherapy for gastric varices have been reported.^9^,^13^-^16^ Treatment related complications were reported far and few between across all cohorts irrespective of differences in etiology of portal hypertension and demographics. The most important factor impacting long time survival and favorable outcomes (negatively) was a high MELD score (>15) or advanced liver disease in worldwide cohorts.^13^-^16^ Such factors were not evaluated in our cohort extensively.

Recurrent bleeding has been the most frequently recorded post procedure complication in all of the aforementioned cohorts; the rates of recurrent bleed reported vary between local and international data, with slightly higher rates of rebleeding encountered in regional centers.^10^,^11^ Recurrent bleed inevitably required another session of sclerotherapy, but results across the board were excellent with nearly complete variceal obliteration and achievement of hemostasis after the second session of N-Butyl 2 Cyanoacrylate injection. Survival rates approaching 95% at five years globally^14^,^15^,^16^ have been reported even after multiple sessions of sclerotherapy with similar short-term outcomes from regional centers; long term data in this regard from loco regional centers is grossly lacking.

Thromboembolic events including pulmonary embolism are recognized post procedural complications of N-Butyl 2 Cyanoacrylate sclerotherapy. But, the incidence of such events previously reported in the literature is extremely low.^15^-^18^ Interestingly enough previous studies have illustrated spontaneous resolution of these events without any intervention at all; infarctions or pulmonary embolism tend to occur within seven days of the procedure and as per reports only require non-specific (symptomatic) treatment.^17^,^18^ With such low incidence rates, pulmonary embolism or any other thromboembolic event for that matter was unlikely to be recorded with a small sample size such as ours; indeed smaller cohorts like ours have previously reported little to zero incidence of thromboembolic events.^19^, ^20^

Our study effectively demonstrated the overall utility of N butyl 2 Cyanoacrylate injection sclerotherapy for the treatment of gastric varices. Our experience and numbers are more akin to local data than international records. This is understandable as differences in stage of liver disease, Child-Pugh score, MELD score and comorbids do tend to affect long term survival. Also, there is geographical variance among the various etiologies of portal hypertension; some are more amenable to treatment than others. How these different factors affect survival beyond bleeding and achievement of hemostasis is certainly a foray for future research.

### Limitations of the study:

Complete nutritional assessment was not performed and regular or non-regular use of Proton pump inhibitors was not assessed. Similarly, use of other medications was neither recorded nor evaluated in patients.

## CONCLUSIONS

N-Butyl 2 Cyanoacrylate injection is safe and effective in treatment of bleeding from gastric varices. It has few side effects which are infrequent and easily dealt with. It reduces mortality across the board. Advanced liver disease, NBNC cirrhosis and co morbids reduce its safety and efficacy.

### Authors’ Contribution:

**NB** conceptualized, conceived, designed and did statistical analysis, writing and editing of the manuscript.

**FH, MAK** did data collection, literature review, analysis and interpretation, statistical analysis and manuscript writing.

**AA** did proof reading, literature review and supervised the study.

**NB** takes the responsibility and accountable for all aspects of the work in ensuring that questions related to the accuracy or integrity of any part of the work are appropriately investigated and resolved.
